# Presumed topiramate retinopathy: a case report

**DOI:** 10.1186/s13256-016-0980-x

**Published:** 2016-08-01

**Authors:** Tiffany L. M. Yeung, Patrick S. H. Li, Kenneth K. W. Li

**Affiliations:** 1 Department of Ophthalmology, United Christian Hospital, 130 Hip Wo Street, Kwun Tong, Kowloon Hong Kong; 2 Department of Ophthalmology, Tseung Kwan O Hospital, 2 Po Ning Lane, Tseung Kwan O, Hong Kong; 3 Department of Ophthalmology, LKS Faculty of Medicine, The University of Hong Kong, Pok Fu Lam, Hong Kong

**Keywords:** Topiramate, Pigmentary retinopathy, Vision disorder, Visual field

## Abstract

**Background:**

We report a case of peripheral pigmentary retinopathy and visual field loss following topiramate use for uncontrolled seizures. Such side effects have not been well documented despite the increasing use of topiramate in the past 10 years. A thorough search of available English literature revealed only a small number of reports of topiramate-induced retinopathy or visual field defects in humans. One similar case has been described. We are concerned about the possible rare instances of this occurrence in future patients and hence would like to propose a presumed correlation.

**Case presentation:**

A 48-year-old Chinese woman developed blurred vision after 9 months of topiramate use. Her visual acuity dropped from 1.2 to 0.7 in both eyes, with bilateral diffuse pigmentary retinopathy and a constricted visual field. Despite an improvement in visual acuity after cessation of the drug, the other clinical findings remained. The temporal relationship between the initiation of topiramate and the visual disturbance suggests that topiramate could be the cause of such signs and symptoms.

**Conclusion:**

Topiramate potentially causes pigmentary retinopathy and constricted visual field.

## Background

Topiramate is a sulfurated drug approved by the US Food and Drug Administration (FDA) for the treatment of partial seizures, migraine, depression, and neuropathic pain. The off-label use of topiramate as a weight-reducing agent has gained popularity. The common dosage used is in the range of 50 to 400 mg per day. Several ocular side effects with explainable mechanisms have been described by the World Health Organization [1], including acute myopia, diplopia, and shallow anterior chamber with angle closure. Other severe ocular side effects were mentioned in previous case reports but the mechanism is not well understood [2]. We report a case of presumed correlation between topiramate and bilateral diffuse pigmentary retinopathy with visual field loss 9 months after initiation of topiramate therapy.

## Case presentation

A 48-year-old Chinese woman with a history of a right parasagittal meningioma with surgery and gamma knife excision done, was first put on valproate but then stepped up to topiramate 26 months later for uncontrolled seizures. The initial dose was topiramate 25 mg twice a day, which was eventually stepped up to 100 mg twice a day for adequate seizure control. She was referred to our ophthalmology clinic for dry eyes before the use of topiramate, with an examination showing a baseline visual acuity of 1.2 in both eyes with unremarkable anterior segment and fundoscopy examination. There was no family history of retinal diseases.

**Fig. 1:**
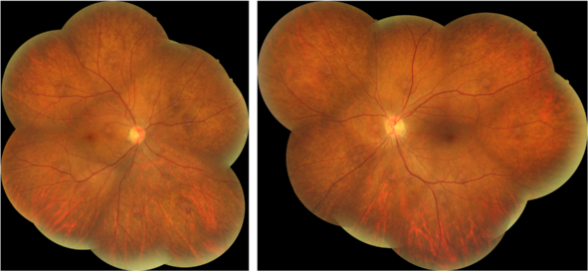
Fundus photo showing bilateral diffuse pigmentary retinopathy

**Fig. 2:**
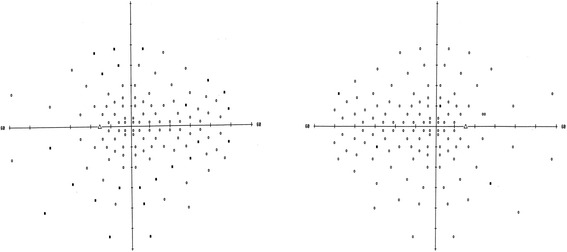
Visual field Full-Field 120 test showing bilateral peripheral constriction

**Fig. 3:**
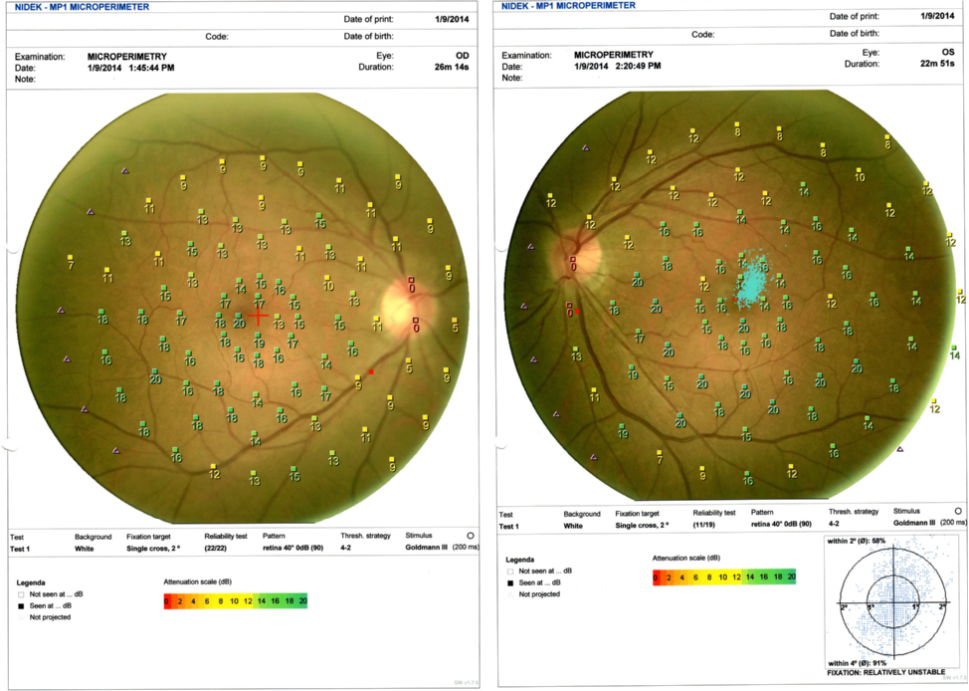
Microperimetry showing normal macula sensitivity

**Fig. 4:**
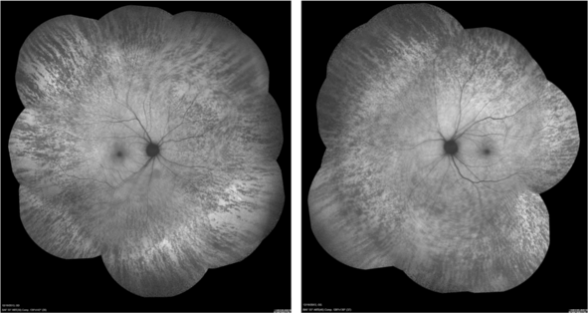
Fundus autofluorescence photos showing bilateral peripheral loss of autofluorescence, compatible with areas with peripheral pigmentary retinopathy

**Fig. 5:**
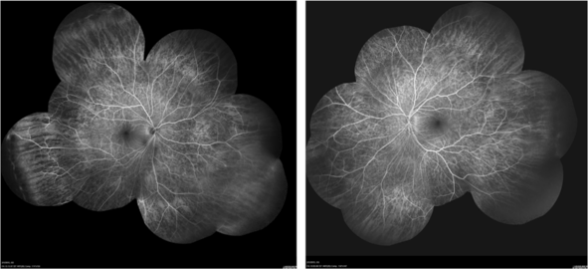
Fundus fluorescein angiogram showing blocked fluorescence in the areas of pigmentation

She complained of blurring of vision in both eyes after using topiramate for 9 months. She was not on other medications when her visual symptoms developed. On examination, her visual acuity was 0.7 in both eyes. Her pupils were equal with no relative afferent papillary defect. The intraocular pressure, Ishihara test, and anterior segment examination were within normal limits. A fundus examination revealed there was bilateral diffuse pigmentary retinopathy (Fig. 1). Automated perimetry showed bilateral peripheral constrictions (Fig. 2) while microperimetry showed normal macula sensitivity (Fig. 3). Autofluorescence fundus pictures showed loss of autofluorescence at the periphery, which is compatible with areas of pigmentary retinopathy (Fig. 4). A fundus fluorescein angiogram also showed blocked fluorescence in the areas of pigmentation (Fig. 5). Optical coherence tomography was unremarkable. A full field electroretinogram (ERG) according to International Society for Clinical Electrophysiology of Vision (ISCEV) standard was performed at 16 months after onset of symptoms (25 months since topiramate was first given). Both photopic and scotopic responses were found to be within normal limits. Administration of topiramate was immediately ceased due to suspected correlation with her eye signs and symptoms. She was switched to levetiracetam monotherapy. There was no more seizure recurrence after this treatment change.

All investigations were repeated 1 year after discontinuation of topiramate. Bilateral diffuse pigmentary retinopathy was still present, with visual field test showing similar peripheral constriction, although her visual acuity improved back to 1.0 in both eyes and she reported subjective improvement in vision.

## Discussion

It has been documented that topiramate can cause ciliochoroidal effusion syndrome, leading to acute myopia, acute angle closure, and macular striae [3]. However, a thorough search of available English literature revealed only a small number of reports of topiramate-induced retinopathy or visual field defects in humans. One similar case has been described [4, 5].

The main visual abnormality found in our patient was bilateral diffuse pigmentary retinopathy and constricted visual field. Without family history of retinal disease, and as our patient was only on topiramate monotherapy while visual blurring occurred, no other risk factors for developing such retinopathy could be identified other than topiramate drug use. We cannot entirely exclude the possibility that the retinal pathology was related to the previous treatment with valproate; however, valproate is a commonly used antiepileptic and no such diseases have been documented in literature. The time course of development of visual impairment after 9 months’ use of topiramate, and the improvement of our patient’s visual acuity after discontinuation of the drug suggests that topiramate is the top etiological factor. Hence, we presume her pigmentary retinopathy with the constricted visual field was caused by topiramate drug toxicity. However, as all structural and functional macular parameters were normal, the reason why there was a visual acuity drop remained unclear. A possible explanation could be that the pigmentary retinopathy was noted and topiramate was immediately stopped at an early stage, when definite signs of maculopathy could not be demonstrated yet.

A proposed mechanism of how topiramate leads to reduction in retinal function has been demonstrated in a rabbit study [6]. Histopathological analyses found extensive accumulation of gamma-aminobutyric acid (GABA) in the inner retina, and ERG found reduction in 30 Hz flicker b-wave amplitude. No human studies have been done so far hence the actual mechanism remains uncertain.

Topiramate is an antiepileptic drug that works on multiple mechanisms, including enhancement of GABA receptor activity. Vigabatrin is another GABAergic antiepileptic drug that is known to cause irreversible visual field defects in more than 30 % of patients [7]. Hence, it is reasonable to postulate that topiramate can cause retinal toxicity similar to that caused by vigabatrin. Further investigation is required before the suspected correlation can be confirmed.

## Conclusions

Topirmate potentially causes pigmented retinopathy and constricted visual field. Although visual acuity returned to normal after immediate cessation of the drug after visual abnormality occurred, ophthalmological findings did not show significant improvement. Hence it is difficult to comment whether or not such pathologies are reversible after discontinuation of drug. Whether the retinopathy is dose-related, whether there is any correlation with duration of treatment, the risk factors for developing such retinopathy, and whether routine screening is necessary, would rely on future studies to answer the above questions.

## Abbreviations

ERG, electroretinogram; FDA, Food and Drug Administration; GABA, gamma-aminobutyric acid; ISCEV, International Society for Clinical Electrophysiology of Vision
